# Analysis of Supportive Evidence for US Food and Drug Administration Approvals of Novel Drugs in 2020

**DOI:** 10.1001/jamanetworkopen.2022.12454

**Published:** 2022-05-17

**Authors:** Mayookha Mitra-Majumdar, Simon J. Gunter, Aaron S. Kesselheim, Beatrice L. Brown, Krysten W. Joyce, Murray Ross, Catherine Pham, Jerry Avorn, Jonathan J. Darrow

**Affiliations:** 1 Program On Regulation, Therapeutics, And Law (PORTAL), Division of Pharmacoepidemiology and Pharmacoeconomics, Department of Medicine, Brigham and Women’s Hospital and Harvard Medical School, Boston, Massachusetts; 2 Kaiser Permanente Institute for Health Policy, Oakland, California; 3 Kaiser Permanente National Pharmacy, Downey, California; 4 Department of Law and Taxation, Bentley University, Waltham, Massachusetts

## Abstract

**Importance:**

In recent years, drug approvals have been based on fewer, smaller, and less rigorous pivotal trials. Less robust preapproval testing raises questions about the efficacy and clinical value of these drugs.

**Objective:**

To assess the regulatory context, pivotal design characteristics, and postmarket requirements (PMRs) and postmarket commitments (PMCs) of novel 2020 drug approvals to characterize the state of evidence at the time of approval.

**Design, Setting, and Participants:**

This cohort study identified novel drugs approved by the US Food and Drug Administration’s (FDA) Center for Drug Evaluation and Research in 2020. The Drugs@FDA database was used to extract key characteristics of each drug’s pivotal trials. Drug approval packages provided regulatory information. The prevalence of key trial design features was compared between oncology and nononcology drugs.

**Exposures:**

Drug names, date of approval, indication on labeling, and clinical and regulatory details.

**Main Outcomes and Measures:**

Number of pivotal trials, pivotal trial design (randomization, masking, groups), trial comparator, trial hypothesis, trial end points, results, number and type of expedited pathway designations, and number and type of PMRs and PMCs.

**Results:**

The 49 novel therapeutics approved in 2020 were supported by 75 pivotal trials. More than half of drugs (28 [57.1%]) were supported by a single pivotal trial. Trial sizes ranged from 19 to 2230 participants. More than three-fourths of trials (57 [76.0%]) had a randomization component, and nearly two-thirds (46 [61.3%]) were double-masked. Most used a superiority approach. Roughly half (39 [52.0%]) compared the novel therapeutic with a placebo or vehicle control; 13 (17.3%), an active control; 2 (2.7%), both a placebo and active control; and 21 (28.0%), a historical, external, or other control. Nearly half of pivotal trials (34 [45.3%]) used a surrogate measure as a primary end point. Pivotal trials supporting oncology approvals were much more likely to have historical controls than nononcology approvals (13 of 18 [72.2%] vs 8 of 57 [14.0%]; *P* < .001) and to use at least 1 surrogate measure as a primary end point (17 [94.4%] vs 17 [29.8%]; *P* < .001). Forty drugs had at least 1 PMR or PMC, accounting for 178 PMRs and PMCs across the cohort.

**Conclusions and Relevance:**

These findings suggest that the increased flexibility in the characteristics of acceptable preapproval evidence can be partially explained by the increase in trials of drugs for rare and other serious conditions that require flexible testing strategies as well as the associated regulatory changes that have accumulated over time. The FDA and consumers may benefit from a revised approach that better balances time to market with ensuring that approved drugs show evidence of efficacy.

## Key Points

**Question:**

What were the key design characteristics of the pivotal trials supporting novel drugs approved by the US Food and Drug Administration (FDA) in 2020?

**Findings:**

This cohort study of 49 drugs approved by the FDA in 2020 found that they were supported by 75 pivotal trials, of which nearly two-thirds were double-masked, more than three-fourths had a randomization component, nearly half used a surrogate measure as a primary end point, and more than one-fourth used a historical, external, or other control. Surrogate measures and historical controls were much more common among oncology drug approvals than among nononcology drug approvals.

**Meaning:**

In this study, the cohort of 2020 novel drug approvals continued a trend of new drugs being supported by smaller numbers of preapproval pivotal trials and fewer features traditionally associated with rigor.

## Teaser

This cohort study assesses the regulatory context, pivotal design characteristics, and postmarket requirements and commitments of novel drugs approved by the US Food and Drug Administration (FDA) in 2020 to characterize the state of evidence at the time of approval.

## Introduction

A principal function of the US Food and Drug Administration (FDA) is to ensure that new drugs have demonstrated benefits that outweigh their risks before the drugs are available for widespread use. In making an approval determination, the FDA is statutorily required to review clinical data submitted by the drug’s sponsor to determine whether there is substantial evidence of the drug’s efficacy from “adequate and well-controlled” trials.^1^ In these regulations, the FDA lists certain features of high-quality clinical trials, such as an appropriate trial comparator, methods promoting valid comparison of the experimental drug to the comparator, and the selection and analysis of a primary outcome measure that satisfactorily illustrates the drug’s efficacy. Further FDA guidance recommends specific design features, such as larger sample sizes, longer participant follow-up periods, and the use of clinical end points to ensure greater certainty of a drug’s clinical benefit. Additionally, it recommends implementing safeguards such as randomization, masking, and placebo control to protect against bias.^2^

However, what constitutes an adequate and well-controlled trial has been the subject of debate in recent years. Reviews of data supporting drug approvals in recent years illustrate the broad discretion that the FDA retains in determining the nature and amount of evidence needed for approval, with standards loosening over time.^3^ The number of drug approvals supported by 2 or more pivotal trials decreased from 81% between 1995 and 1997 to 53% between 2015 and 2017.^3^ Use of double-masking decreased from 80% to 68%, randomization from 94% to 82%, and active comparators from 44% to 29%.^3^ The nature of the end points used in drug approvals has also come under scrutiny, as the FDA has increasingly accepted surrogate measures, such as laboratory tests and biomarkers, in place of more direct measurements of a participant’s clinical status, such as how a patient feels, functions, or survives. Nearly 60% of drugs approved between 2015 and 2017 were supported by surrogate measures compared with 44% of drugs approved between 2005 and 2012.^3^ Part of this shift has been driven by developments in certain therapeutic classes; for example, smaller trials based on biomarkers or surrogate measures have been more common and accepted in oncology.^4^

The growth in the use of surrogate measures has coincided with the introduction of new regulatory pathways at the FDA that intentionally facilitate the approval of new drugs based on fewer, smaller, or less rigorous pivotal trials, with the goal of reducing drug development costs and promoting the expedited approval of more new drugs.^5,6^ While this may yield more approved drugs, the benefit of these drugs for patients may be more uncertain on approval, with the burden of evidence collection shifted to the postapproval period.^7^ For example, the FDA initiated the Accelerated Approval pathway in 1992 to enable the approval of drugs for serious conditions based on surrogate, rather than clinical, end points.^8^ Studies have found that drugs granted Accelerated Approval are more likely to be approved on fewer, smaller, and shorter clinical trials. Rigorous postapproval trials to confirm efficacy are critical in these cases, yet both preapproval and postapproval confirmatory trials commonly used nonrandomized designs with a reliance on surrogate end points.^9^ Less rigorous preapproval trials foster uncertainty about a drug’s efficacy and safety.^10^

These changes in the regulatory landscape raise questions about the nature of evidence underlying recent drug approvals and the extent to which the clinical value of each new drug is robustly characterized at the time of approval. In this article, we investigate the evidence supporting recent drug approvals, focusing on the characteristics of pivotal trials (the primary trials the FDA relies on for approval), expedited approval pathways, and the number and nature of any postapproval data collection requirements.

## Methods

This cohort study examined all novel FDA drug approvals in 2020. We performed this search in March 2021. Our study used data that was publicly available and did not include human participant research. As per 45 CFR §46.102(f), this study was not submitted for institutional review board approval and did not require informed consent procedures. We adhered to the Strengthening the Reporting of Observational studies in Epidemiology (STROBE) reporting guidelines.

We used the FDA’s website to identify small-molecule drugs and therapeutic biologics approved by the FDA’s Center for Drug Evaluation and Research in 2020. We excluded imaging agents because their purpose is diagnostic, rather than therapeutic, and the structure of their pivotal trials differs meaningfully from that of therapeutic agents. We also excluded new generic drugs and any products approved by the FDA’s Center for Biologics Evaluation and Research (such as vaccines, biosimilars, allergenic products, blood and blood products, and cellular and gene therapies). Also excluded were pivotal trials supporting expanded or different indications (eg, new populations, diseases, dosages, or formulations) of already approved drugs. For each drug, we extracted key characteristics of the pivotal trials from approval documentation available at the Drugs@FDA website, including the number of trial participants; trial design features, such as randomization, masking, and number of groups; the use of an active, placebo, or historical, external, or other control; whether the trial was designed to demonstrate superiority or noninferiority to a comparator; whether the primary end point, an outcome measure that reflects laboratory findings, symptoms, or other signs of disease states, is a surrogate or clinical measure; and trial results.^11^

We characterized comparators as active, placebo, placebo and active, and historical, external, or other. While several pivotal trials included both a placebo and active comparator, we only characterized the trial as both if the primary end point specified comparisons to both the placebo and active comparator. We typically grouped trials with a vehicle control with trials using a placebo control. A vehicle control is the substance in which the experimental treatment is formulated but without the active ingredient and is a typical control for topical drugs. Furthermore, we grouped single-group trials into the historical, external, or other comparator category, even when an explicitly historical or natural control was not stated. This is in line with how the FDA has viewed single-group trials.^12^ Finally, clinical end points measure how a patient functions, feels, or survives, while surrogate end points are laboratory measurements believed to correlate with changes in clinical end points.^13^ We also divided our data set into drugs for oncology indications and those for nononcology indications.

We next extracted approval packages from Drugs@FDA as well as press releases from FDA and drug sponsors to identify any special FDA designations, including Orphan Drug Act designation for drugs treating rare diseases, fast track, accelerated approval, priority review, and breakthrough therapy. Finally, we used each drug’s approval package to collect postmarket requirements (PMRs) and postmarket commitments (PMCs), the 2 types of postapproval studies that the FDA can seek at the time of drug approval. While PMCs are agreements made by the manufacturer at the time of approval and are reported under the Food, Drug, and Cosmetic Act (FDCA) §506B, PMRs are required by statute. It has been proposed that more flexible preapproval evidentiary standards shift evidence generation to the postapproval period via PMRs and PMCs.

We categorized whether the PMRs were mandated under Accelerated Approval, as a follow up for drugs approved based on a certain kind of surrogate measure; the Animal Efficacy Rule, for human testing of a drug approved based only on animal studies; the Pediatric Research Equity Act (PREA), to test newly approved drugs in children; or Food and Drug Administration Amendments Act (FDAAA) §505(o)(3) to investigate a range of safety and efficacy issues. We included reportable PMCs under FDCA §506B but excluded nonreportable PMCs from our data collection and analysis. Nonreportable PMCs are primarily focused on chemistry, manufacturing, and control studies related to product quality. We assessed the PMRs and PMCs more likely to be relevant to efficacy and safety evaluations.

### Statistical Analysis

For oncology and nononcology drug approvals, we computed summary statistics at the trial and drug level on randomization, double-masking, use of surrogate measures, and trial comparators. We then used SAS version 9.4 (SAS Institute) to run χ^2^ tests to determine whether differences the 2 groups were statistically significant at the .05 level.

## Results

The FDA approved 49 novel therapeutics in 2020, higher than the annual mean of 40 drugs between 2011 and 2020. We collected the brand name, generic name, drug sponsor, date of approval, and indication for all drugs (Table 1). Of these, 36 (73.5%) were small-molecule drugs approved via New Drug Applications, and the remainder (13 [26.5%]) were biologics approved via Biologics License Applications.

**Table 1.** Novel Drugs Approved by the US Food and Drug Administration in 2020^a^

Drug	Brand name	Approval date	Sponsor	Indication
Avapritinib	Ayvakit	01/09/20	Blueprint Medicines	Treatment of unresectable or metastatic gastrointestinal stromal tumor
Teprotumumab-trbw	Tepezza	01/21/20	Horizon Therapeutics Ireland	Treatment of thyroid eye disease
Tazemetostat	Tazverik	01/23/20	Epizyme	Epithelioid sarcoma
Lactitol	Pizensy	02/12/20	Braintree Labs	Treatment of chronic idiopathic constipation
Bempedoic acid	Nexletol	02/21/20	Esperion Therapeutics	Treatment of heterozygous familial hypercholesterolemia or established atherosclerotic cardiovascular disease
Eptinezumab-jjmr	Vyepti	02/21/20	Lundbeck Seattle BioPharmaceuticals	Preventive treatment of migraines
Amisulpride	Barhemsys	02/26/20	Acacia	Preventive treatment of postoperative nausea and vomiting
Rimegepant	Nurtec ODT	02/27/20	Biohaven Pharmaceuticals	Treatment of migraines
Isatuximab-irfc	Sarclisa	03/02/20	Sanofi Aventis US	Treatment (adjunctive therapy) of refractory multiple myeloma
Osilodrostat	Isturisa	03/06/20	Novartis	Treatment of Cushing disease in which pituitary gland surgery has failed or is contraindicated
Ozanimod	Zeposia	03/25/20	Celgene	Treatment of relapsing forms of multiple sclerosis
Selumetinib	Koselugo	04/10/20	AstraZeneca	Treatment of neurofibromatosis type 1
Tucatinib	Tukysa	04/17/20	Seattle Genetics	Treatment (adjunctive therapy) of advanced unresectable or metastatic *ERBB2*-positive breast cancer
Pemigatinib	Pemazyre	04/17/20	Incyte	Treatment of locally advanced or metastatic cholangiocarcinoma
Sacituzumab govitecan-hziy	Trodelvy	04/22/20	Immunomedics	Treatment of metastatic triple-negative breast cancer
Opicapone	Ongentys	04/24/20	Neurocrine Biosciences	Treatment (adjunctive therapy) of “off” episodes in Parkinson disease
Capmatinib	Tabrecta	05/06/20	Novartis	Treatment of non–small cell lung cancer
Selpercatinib	Retevmo	05/08/20	Loxo Oncology	Treatment of metastatic non–small cell lung cancer and thyroid cancers
Ripretinib	Qinlock	05/15/20	Deciphera Pharmaceuticals	Treatment of gastrointestinal stromal tumor
Artesunate	Artesunate	05/26/20	Amivas	Treatment of severe malaria
Inebilizumab-cdon	Uplizna	06/11/20	Viela Biopharmaceuticals	Treatment of neuromyelitis optica spectrum disorder
Lurbinectedin	Zepzelca	06/15/20	Pharma Mar USA	Treatment of metastatic small cell lung cancer
Triheptanoin	Dojolvi	06/30/20	Ultragenyx Pharmaceuticals	Treatment of long-chain fatty acid oxidation disorders
Remimazolam	Byfavo	07/02/20	Acacia	Induction and maintenance of procedural sedation
Fostemsavir	Rukobia	07/02/20	Viiv Healthcare Company	Treatment (adjunctive therapy) of HIV-1
Decitabine and cedazuridine	Inqovi	07/07/20	Astex Pharmaceuticals	Treatment of myelodysplastic syndromes
Abametapir	Xeglyze	07/24/20	Dr Reddy’s Laboratories	Treatment of head lice
Tafasitamab-cxix	Monjuvi	07/31/20	MorphoSys US	Treatment (adjunctive therapy) of relapsed or refractory diffuse large B-cell lymphoma
Belantamab mafodotin-blmf	Blenrep	08/05/20	GlaxoSmithKline	Treatment of relapsed or refractory multiple myeloma
Nifurtimox	Lampit	08/06/20	Bayer Healthcare	Treatment of Chagas disease in pediatric patients younger than 18 years
Risdiplam	Evrysdi	08/07/20	Genentech	Treatment of spinal muscular atrophy
Oliceridine	Olinvyk	08/07/20	Trevena	Management of severe acute pain
Viltolarsen	Viltepso	08/12/20	Nippon Shinyaku	Treatment of Duchenne muscular dystrophy
Satralizumab-mwge	Enspryng	08/14/20	Genentech	Treatment of neuromyelitis optica spectrum disorder
Clascoterone	Winlevi	08/26/20	Cassiopea SpA	Treatment of acne vulgaris
Somapacitan-beco	Sogroya	08/28/20	Novo Nordisk	Treatment of growth hormone deficiency
Pralsetinib	Gavreto	09/04/20	Genentech	Treatment of non–small cell lung cancer
Atoltivimab, maftivimab, and odesivimab-ebgn	Inmazeb	10/14/20	Regeneron	Treatment of Ebola virus
Remdesivir	Veklury	10/22/20	Gilead Sciences	Treatment of COVID-19
Lonafarnib	Zokinvy	11/20/20	Eiger BioPharmaceuticals	Reduction of mortality risk in rare genetic diseases that cause premature aging
Lumasiran	Oxlumo	11/23/20	Alnylam Pharmaceuticals	Treatment of primary hyperoxaluria type 1
Setmelanotide	Imcivree	11/25/20	Rhythm Pharmaceuticals	Treatment of obesity and hunger control due to proopiomelanocortin deficiency
Naxitamab-gqgk	Danyelza	11/25/20	Y-mAbs Therapeutics	Treatment (adjunctive therapy) of refractory or relapsed high-risk neuroblastoma
Berotralstat	Orladeyo	12/03/20	BioCryst Pharmaceuticals	Preventive treatment for attacks of hereditary angioedema
Tirbanibulin	Klisyri	12/14/20	Athenex	Treatment of actinic keratosis of the face or scalp
Margetuximab (anti-*ERBB2* mAb	Margenza	12/16/20	MacroGenics	Treatment of metastatic *ERBB2*-positive breast cancer
Relugolix	Orgovyx	12/18/20	Myovant Sciences	Treatment of advanced prostate cancer
Ansuvimab-zykl	Ebanga	12/21/20	Ridgeback Biotherapeutics	Treatment of Ebola virus
Vibegron	Gemtesa	12/23/20	Urovant Sciences	Treatment of overactive bladder

^a^ Excludes 4 diagnostic agents that were approved by the FDA in 2020.

### Masking, Randomization, Trial Groups, and Enrollment

A total of 75 pivotal trials supported the 49 drugs approved in 2020, with 28 drugs (57.1%) approved on the basis of a single pivotal trial, 17 (34.7%) on the basis of 2, and 4 (8.2%) on the basis of 3 or more (range, 1-4). Three-fourths (57 [76.0%]) had a randomization component, and 61.3% (46) were double-masked. Most pivotal trials had 2 groups (46 [61.3%]), but nearly one-quarter (18 [24.0%]) were single-group trials. Trial sizes ranged from 19 to 2230 participants.

### Trial Comparator, Hypothesis, and Primary End Point

More than half of pivotal trails (39 [52.0%]) used a placebo or vehicle control, 13 (17.3%) used an active control, 2 (2.7%) had both a placebo and active control, and 21 (28.0%) used a historical, external, or other control. Frequently, the trial’s primary end point was in reference to the placebo group. Sixteen of the 25 drugs (64.0%) approved with 2 or more pivotal trials featured the same comparator across all pivotal trials. For example, eptinezumab-jjmr (for the preventive treatment of migraines) had 2 pivotal trials, both of which used a placebo control. In the remaining 9 instances, multiple pivotal trials supporting approval either used different comparators or used study designs meant to investigate dose or length of treatment rather than efficacy relative to alternatives.

Most pivotal trials (66 [88.0%]) used a superiority approach. Most of the remainder were noninferiority trials, which are typically used with active controls and attempt to demonstrate that the investigational drug is noninferior to the active control.^14^ A smaller portion were equivalence trials. Pivotal trials are powered to detect anticipated differences in the primary end points, which can be either clinical or surrogate measures.^15^ Nearly half of all pivotal trials (34 [45.3%]) used a surrogate measure as the primary end point, and more than half of all drug approvals (28 [57.1%]) were based on at least 1 pivotal trial using a surrogate measure.

### Variation by Therapeutic Class

One-third of drugs (17 of 49 [34.7%]) were for oncology indications, including 4 for non–small cell lung cancers—capmatinib, selpercatinib, lubinectedin, and pralsetinib—and 3 other drugs for various types of breast cancer—tucatinib, sacituzumab govitecan-hziy, and margetuximab. The 17 oncology drugs accounted for 18 pivotal trials; the 32 nononcology drugs accounted for 57 pivotal trials. Pivotal trials supporting oncology approvals were much more likely to use a historical control than nononcology approvals (13 [72.2%] vs 8 [14.0%]; *P* < .001) and less likely to use a placebo or vehicle control (1 [5.6%] vs 38 [66.7%]; *P* < .001) (Table 2). Drugs for oncology indications, compared with those for nononcology indications, also had fewer pivotal trials with a randomized component (6 [35.3%] vs 30 [93.8%]; *P* < .001) and were more likely to have been approved based on at least 1 surrogate primary end point (16 [94.1%] vs 12 [37.5%]; *P* < .001). For example, isatuximab-irfc was approved as an adjunctive therapy for multiple myeloma in treatment-experienced patients based on a single pivotal trial. The primary end point in the pivotal trial was progression-free survival (PFS), defined as the time from randomization to the date of first documentation of progressive disease as determined by the study’s independent review committee. PFS is an established surrogate measure for overall survival from other multiple myeloma studies.^16^

**Table 2.** Summary Statistics for All 2020 Drug Approvals Compared With Oncology Subset^a^

Characteristic	2020 Drug approvals	2020 Nononcology approvals	2020 Oncology approvals	*P* value^b^
Total drugs, No.	49	32	17	NA
Total pivotal trials, No.	75	57	18	NA
Pivotal trials per drug, median (range)	1 (1-4)	2 (1-4)	1 (1-2)	NA
Design characteristics, by trial, No./total No. (%)				
Partial or complete randomization	57/75 (76.0)	51/57 (89.5)	6/18 (33.3)	<.001
Double masking	46/75 (61.3)	44/57 (77.2)	2/18 (11.1)	<.001
Surrogate end point	34/75 (45.3)	17/57 (29.8)	17/18 (94.4)	<.001
Design characteristics, by drug, No./total No. (%)				
Randomization	36/49 (73.5)	30/32 (93.8)	6/17 (35.3)	<.001
Double masking	28/49 (57.1)	26/32 (81.3)	2/17 (11.8)	<.001
Surrogate end point	28/49 (57.1)	12/32 (37.5)	16/17 (94.1)	<.001
Comparator, by trial				
Placebo or vehicle control	39/75 (52.0)	38/57 (66.7)	1/18 (5.6)	<.001
Active control	13/75 (17.3)	10/57 (17.5)	3/18 (16.7)	.93
Placebo and active control	2/75 (2.7)	1/57 (1.8)	1/18 (5.6)	.38
Historical, external, or other control	21/75 (28.0)	8/57 (14.0)	13/18 (72.2)	<.001

Abbreviation: NA, not applicable.

^a^ Cohort excludes 4 novel diagnostic agents approved by the US Food and Drug Administration in 2020.

^b^ The χ^2^ test was used to compare the differences between the nononcology and oncology approval groups.

### Expedited FDA Approval Pathways

Thirty-nine drugs (73.5%) qualified for at least 1 of the 5 special FDA development or review designations, with 25 (51.0%) qualifying for 3 or more (Table 3). The most common designations were priority review (28 [57.0%]) and Orphan Drug Act designation (30 [61.2%]). The least common was accelerated approval, granted to just 12 drugs (24.5%).

**Table 3.** Special FDA Designations for 2020 Novel Drug Approvals

Designation	Eligibility	Benefit	Drugs, No. (%) (N = 49)	Examples
Priority review	Drugs that may significantly improve safety or effectiveness of the treatment, diagnosis, or prevention of serious conditions	Action taken on an NDA or BLA within 6 mo, rather than the standard 10 mo	28 (57)	Tazmetostat (Tazverik) Relugolix (Orgovyx)
Accelerated approval	Drugs that would treat serious conditions and fill an unmet medical need	Approval based on a surrogate or intermediate clinical end point, with required phase 4 confirmatory trials	12 (25)	Tafasitamab-cxix (Monjuvi) Nifurtimox (Lampit)
Fast track	Drugs that would treat serious conditions and fill an unmet medical need	Drug entitled to additional FDA meetings and input on trial design; rolling review; and eligibility for accelerated approval and priority review if certain criteria are met	16 (33)	Teprotumumab-trbw (Tepezza) Margetuximab (Margenza)
Breakthrough therapy	Drugs that would treat serious conditions and for which preliminary clinical evidence indicates improvement over available therapies on clinically significant end point(s)	Drug entitled to all aspects of fast track, intensive guidance on efficient drug development, and high-level organizational commitment from FDA	22 (45)	Artesunate (Artesunate) Ansuvimab-zykl (Ebanga)
Orphan drug	Drugs that treat diseases qualifying for orphan status, defined by 21 CFR Part 316 as those affecting fewer than 200 000 people in the US	Tax credits for qualified clinical testing and a waiver for the prescription drug user fee (unless the application includes a nonorphan indication)	30 (61)	Osilodrostat (Isturisa) Triheptanoin (Dojolvi)

Abbreviations: BLA, biologics license application; FDA, US Food and Drug Administration; NDA, new drug application.

### PMRs and PMCs

A total of 178 PMRs and PMCs were associated with the 49 drugs approved in 2020, a mean (SD) 3.6 (3.1) per drug (Table 4). Of the 49 drugs, 40 (81.6%) had 1 or more PMRs and/or PMCs, while 9 (18.4%) had none. Among drugs with at least 1 PMR or PMC, there was mean (SD) of 4.5 (2.9) per drug. Among all PMRs and PMCs, thirteen (7.3%) were required under accelerated approval regulations, 35 (19.7%) under PREA, 95 (53.4%) under FDAAA §505(o)(3), and 35 (19.7%) under FDCA §506B. Among the 40 drugs with PMRs and PMCs, 12 (30.0%) had at least 1 accelerated approval PMR, 12 (30.0%) had a PREA PMR, 30 (75.0%) had a FDAAA §505(o)(3) PMR, and 18 (45.0%) had a PMC under FDCA §506B.

**Table 4.** PMRs and PMCs for 2020 Drug Approvals^a^

Authority (year enacted)	Description	PMRs and PMCs for drugs approved in 2020, No. (%) (N = 178)	Drugs approved in 2020 with PMRs and PMCs No. (%) (N = 40)
Accelerated approval (1992)	Studies and clinical trials to verify the clinical benefit of drugs approved based on surrogate end point(s)	13 (7.3)	12 (30)
Animal Efficacy Rule (2002)	Studies and clinical trials intended to verify and describe the drug’s clinical benefit in humans when the drug has been approved on animal studies and other supporting data	0	0
Pediatric Research Equity Act (2003)	Studies and clinical trials meant to improved quality of pediatric information in drug labeling; may be waived under certain conditions	35 (19.7)	12 (30.0)
FDAAA §505(o)(3) (2007)	Studies and clinical trials meant to assess a known serious risk related to the use of a drug, signals of serious risk related to the use of the drug, or identify an unexpected serious risk when available data indicate potential for serious risk	95 (53.4)	30 (75.0)
Reportable PMCs under Food, Drug, and Cosmetic Act §506B	Other studies and clinical trials agreed to by the sponsor	35 (19.7)	18 (45)

Abbreviations: FDAAA, Food and Drug Administration Amendments Act; PMC, postmarket commitment; PMR, postmarket requirement.

^a^ Of the 49 drugs in our sample, 40 had at least 1 PMR or PMC associated; 9 had no PMRs or PMCs.

## Discussion

Our study found that the evidence quality underlying new drug approvals in 2020 reflects a trend consistent with those observed in prior analyses. Of the pivotal trials supporting the 49 novel therapeutics approved in 2020, three-fourths featured a randomization component and roughly two-thirds were double-masked. But some characteristics raised questions about the strength of the supporting evidence, such as the finding that 28.0% relied on a historical, external, or other control rather than a concurrent placebo, vehicle, or active control group. Nearly half of all pivotal trials used a surrogate measure. PMRs and PMCs were common and take on increased importance in cases in which they are intended to fill gaps in efficacy and safety left by preapproval evidence limited in amount and/or quality.

Inconsistencies in the amount and quality of evidence supporting new drugs can be explained by several factors. Drugs for rare diseases and other serious conditions have become more common and may necessitate flexible testing strategies. The FDA classified 19 of the 49 therapeutics (38.8%) as first-in-class. First-in-class drugs typically have a new mechanism of action and are generally considered to be more innovative.^4^ First-in-class drugs addressing unmet medical need, as well as gene therapies and other precision medications, may also require flexible strategies in clinical trials. More than 60% of 2020 new therapeutic approvals (30 [61.2%]) were granted Orphan Drug Act designation. Orphan drugs treat diseases that affect fewer than 200 000 people in the United States. Existing therapies are less likely, potentially precluding the use of an active control. As the FDA has explained, patients with severe illness and without other choices “are generally willing to accept greater risks,”^17^ providing a rationale for the approval of drugs based on more uncertain evidence.

For example, risdiplam was approved in 2020 to treat patients aged 2 months and older with spinal muscular atrophy (SMA), a very rare disease.^18,19^ It is only the third drug to treat SMA since nusinersen (approved 2016) and onasemnogene abeparvovec-xioi (approved 2019) were approved. The 2 pivotal trials for risdiplam enrolled a total of 201 patients, used a nonrandomized design, and had 2 separate surrogate measures. The small number of patients with SMA and recently approved treatment options may help to explain these design elements of risdiplam’s pivotal trials. Placebo control has sometimes been considered unacceptable for severe disease, and small numbers of patients could result in insufficient statistical power to detect modest differences in efficacy if patients were randomized.^20^

Accumulated changes to the FDA’s evaluation framework, including the initiation of expedited approval programs, have enabled a broader range of evidence supporting new drugs.^21^ For example, in 1997, Congress formally codified the FDA’s existing practice of accepting a single trial as adequate to support approval in some circumstances.^22^ In the 2012 FDA Safety and Innovation Act, Congress encouraged increased use of surrogate measures and “fewer, smaller, and shorter”^23^ clinical trials. In the 2016 21st Century Cures Act, Congress directed the FDA to update or issue guidance explaining how “adaptive and other novel trial designs”^24^ could satisfy the legal standard for approval. These efforts aim to accelerate the development and approval of drugs for serious conditions. Increasing flexibility in evidence standards may shorten development time, lower costs, and advance the start of drug companies’ revenue streams. Taken together, these may induce drug companies to pursue development programs that might otherwise be less attractive, potentially facilitating more drugs to market over time. As our study found, 2020 yielded the highest number of new drug approvals in a single year in the last 3 decades, with the exception of 2018 (59 drugs) and 1996 (60 drugs).^6^

However, reduced evidence requirements for marketing authorization raise concerns about the impact of the drugs on patient outcomes. First, preliminary findings from phase 2 trials showing large effect sizes sometimes have not been duplicated in phase 3 trials.^25^ Second, while some surrogate measures may be validated—meaning that studies have demonstrated a correlation between the surrogate and a clinical outcome of interest—others are less well established^26^ and correlate poorly with clinical outcomes, particularly in oncology.^27^ One study^28^ found that treatment effects of 5 surrogate measures commonly used in clinical trials for breast cancer drugs, such as pathological complete response rates, were weakly correlated with treatment effects on overall survival. Drugs in the accelerated approval pathway, which allows certain drugs for serious conditions to be approved based on surrogate measures that are not yet well established, require postapproval confirmatory studies. However, studies have shown that many postapproval confirmatory studies for accelerated approval drugs still use surrogate instead of direct measures.^9^ Increasingly, pivotal trials for drugs approved via regular approval, particularly in oncology, are also using surrogate measures, without the same requirement for postapproval confirmatory studies.^29^ The shift from direct clinical end points to surrogates over time decreases confidence that these drugs improve patient outcomes.

Third, allowing a lower quantity or quality of evidence for FDA approval typically defers evidence generation to the postapproval period through PMRs and PMCs. While this strategy may expedite access to necessary medications, potential negative consequences include approval of low-value drugs and the occurrence of adverse safety events not identified during limited preapproval studies. It is also unclear the degree to which the FDA is able to ensure timely completion of PMRs and PMCs, despite the FDA’s assertion that limited preapproval evidence can be supplemented from evidence of more rigorous ongoing studies, PMRs and PMCs, and other evidence from routine clinical use.^30,31^ The expectation is that evidence generated through PMRs and PMCs will quickly inform clinical practice, but this has not occurred consistently in the past.^31^

### Limitations

There are 2 main limitations of this study. First, we conducted a descriptive analysis interrogating whether a recent year of drug approvals demonstrated trends in line with previous years, but we did not conduct a formal statistical trend comparison. The small sample size of drugs limits the value of subanalyses by indication, expedited approval pathways, and other defining characteristics. Second, while we noted the significant number of PMRs and PMCs associated with 2020 novel drug approvals, the study cohort was too recent to assess their outcomes. An in-depth follow-up, using methods set by published studies of earlier time periods, could investigate whether these PMRs and PMCs would answer questions left by limited preapproval evidence, timeliness of completion, and whether the FDA takes appropriate action based on the results of PMRs and PMCs.^32,33,34^

## Conclusions

In this study, 2020 drug approvals illustrated varying levels of clinical trial rigor based on our analysis of the number, design, and findings of pivotal trials. Roughly three-quarters received at least 1 special regulatory designation. Preapproval evidence for these drugs was intended to be supplemented with evidence from a total of 178 PMRs and PMCs. Because they are ongoing or have not yet begun, we cannot yet draw conclusions related to findings and timeliness of these studies. The pivotal trials of oncology drugs approved in 2020 had more flexible trial designs than approvals overall, including randomization, use of surrogate end points, and use of a historical control. Without a reasonable likelihood that these drugs provide substantial clinical benefits, the argument for more rapid entry becomes less compelling.^35^ The FDA and consumers may benefit from a reexamination of how the agency balances time to market with ensuring that approved drugs are sufficiently safe and effective.
